# Gastric extremely well differentiated adenocarcinoma of gastric phenotype: as a gastric counterpart of adenoma malignum of the uterine cervix

**DOI:** 10.1186/1477-7819-3-28

**Published:** 2005-05-23

**Authors:** Won Ae Lee

**Affiliations:** 1 Department of pathology, College of Medicine Dankook University, Cheonan, Republic of Korea

## Abstract

**Background:**

Most of gastric adenocarcinoma can be simply diagnosed by microscopic examination of biopsy specimen. Rarely the structural and cellular atypia of tumor cells is too insignificant to discriminate from benign foveolar epithelium.

**Case presentation:**

A 67-year-old male presented with a gastric mass incidentally found on the abdominal computed tomography (CT) for routine medical examination. Gastric endoscopic examination revealed a huge fungating mass at the cardia and mucosal biopsy was performed. Microscopically the biopsy specimen showed proliferation of bland looking foveolar epithelia in the inflammatory background and diagnosed as foveolar epithelial hyperplasia. Because the clinical and endoscopic features of this patient were strongly suggestive of malignancy, the patient underwent radical total gastrectomy. The resected stomach revealed a huge fungating tumor at the cardia. The cut surface of the tumor was whitish gelatinous. Microscopically the tumor was sharply demarcated from surrounding mucosa and composed of very well formed glandular structures without significant cellular atypia, which invaded into the whole layer of the gastric wall. Tumor glands were occasionally complicated or dilated, and glandular lumina were filled with abundant mucin. Immunohistochemically the tumor cells revealed no overexpression of p53 protein but high Ki-67 labeling index. The tumor cells and intraluminal mucin were diffusely expressed MUC1 and MUC5AC and only focally expressed MUC2. On abdominal CT taken after 12 months demonstrated peritoneal carcinomatosis and multiple metastatic foci in the lung.

**Conclusion:**

The clinicopathologic profiles of gastric extremely well differentiated adenocarcinoma of gastric phenotype include cardiac location, fungating gross type, very similar histology to foveolar epithelial hyperplasia, foveolar mucin phenotype, lack of p53 overexpressoin and high proliferative index.

## Background

Most of gastric adenocarcinoma can be simply diagnosed by microscopic examination of biopsy specimen. Extremely well differentiated adenocarcinoma (EWDA) of the stomach is histologically too bland and too similar to benign foveolar epithelium to make a diagnosis as malignancy. Till date, several cases of EWDA of the stomach were reported by Japanese authors. But the cases reported as EWDA were heterogeneous groups histologically and phenotypically. Most of reported cases revealed well differentiated adenocarcinoma mimicking complete type intestinal metaplasia with intestinal mucin phenotype [1,2]. Only few cases corresponded to EWDA of the stomach mimicking reactive foveolar epithelia with gastric mucin phenotype [3]. Recently I experienced a case of EWDA of the stomach which was very similar to benign foveolar epithelia histologically and phenotypically and reminiscent of gastric counterpart of adenoma malignum of the uterine cervix [4].

## Case presentation

**Figure 1:**
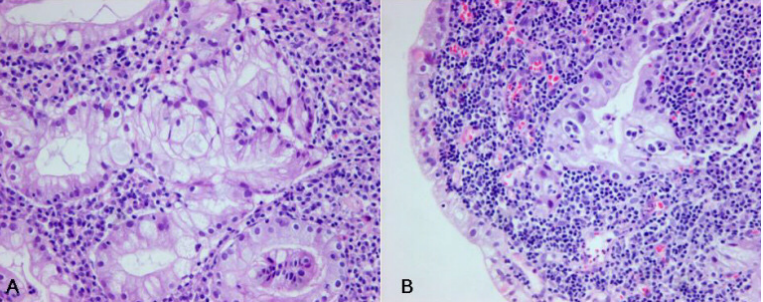
**Microscopic photograph of the biopsied specimen**. A. Bland glands mimicking benign foveolar epithelial hyperplasia are noted in the heavy inflammatory backgrounds (hematoxylin and eosin, X400). B. Glands are destructed by inflammatory cell infiltrates and epithelial cells reveal mild atypia (hematoxylin and eosin, X400).

**Figure 2:**
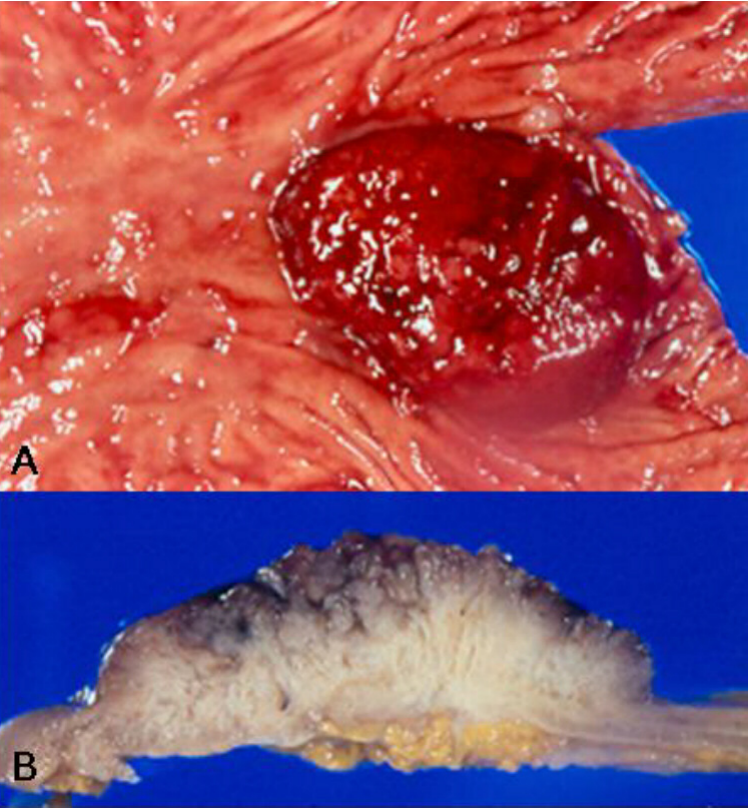
**Macroscopic photograph of the tumor**. A. Resected stomach reveals huge fungating tumor at the cardia. B. The cut surface of the tumor is whitish gelatinous and the tumor involves the whole layer of the gastric wall.

**Figure 3:**
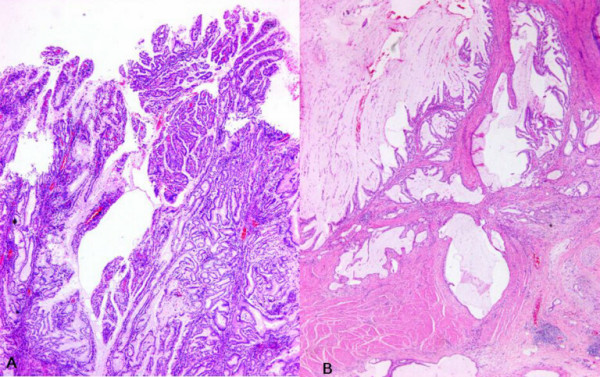
**Low power view of microscopic photograph of the tumor**. A. Tumor glands proliferate haphazardly with papillary configuration at the surface (hematoxylin and eosin, X40). B. Cystically dilated glands invade to proper muscle and subserosa (hematoxylin and eosin, X40).

**Figure 4:**
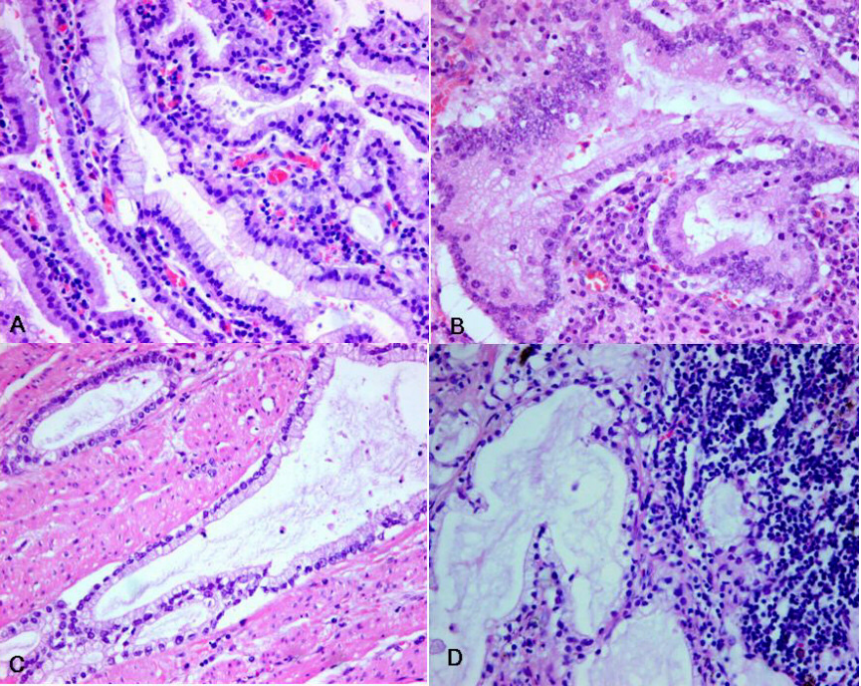
**High power view of microscopic photograph of the tumor**. A. Bland glands are lined by mucin-rich columnar cells with basal nuclei (hematoxylin and eosin, X400). B. More complicated glands are lined by more atypical nuclei with prominent nucleoli (hematoxylin and eosin, X400). C. Very benign looking glands are present within muscle (hematoxylin and eosin, X400). D. Metastatic tumor glands within lymph node show insignificant cellular atypia (hematoxylin and eosin, X400).

**Figure 5:**
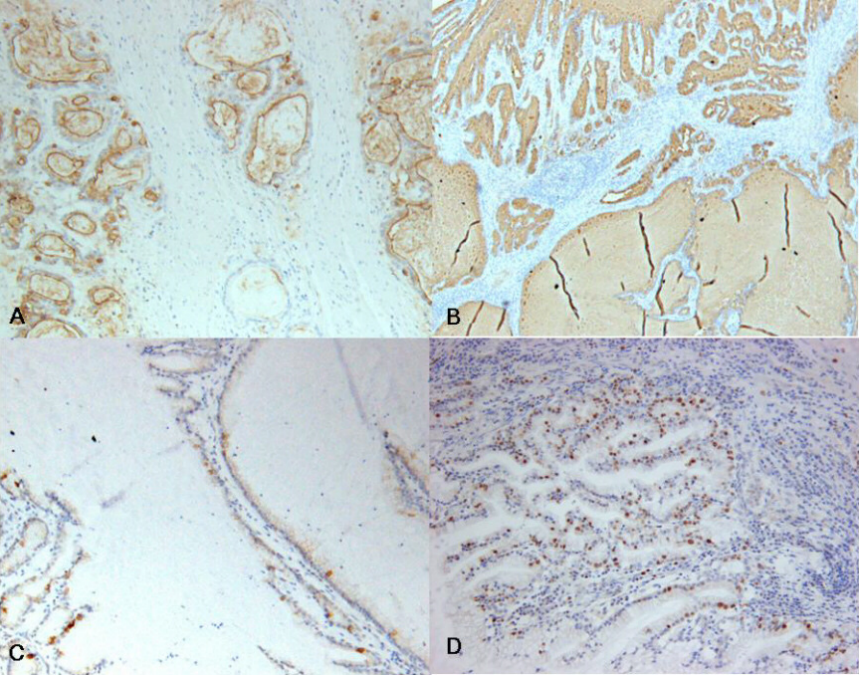
**Immunohistochemical finding of the tumor**. The tumor cells and intraluminal mucin are diffusely expressed MUC1 (A) and MUC5AC (B). MUC2 expression is focally noted in some tumor cells (C). Tumor cells reveal high Ki-67 labeling index (D). (Immunohistochemistry, X100).

A 67-year-old male presented with a gastric mass incidentally found on the abdominal computed tomography (CT) for routine medical examination. Abdominal CT showed a fungating tumor at the gastric cardia and several lymph node enlargements at the left gastric and celiac axis. Gastric endoscopic examination revealed a huge fungating mass at the cardia, and subsequently mucosal biopsy was performed. Microscopically the biopsy specimen showed proliferations of bland looking hyperplastic foveolar epithelia with basally located small nuclei and fine nuclear chromatin in the heavy inflammatory background (Figure 1A). Some glands were destructed by inflammatory cell invasion and revealed mild epithelial atypia with mildly increased nuclei and loss of nuclear polarity reminiscent of reactive cellular atypia (Figure 1B). This biopsy specimen was diagnosed as foveolar epithelial hyperplasia. However, the clinical and endoscopic features of this patient were strongly suggestive of malignancy. The patient underwent radical total gastrectomy with *Roux en Y *anastomosis. The extent of lymph node dissection included first and second lymph node groups. The resected stomach revealed a huge fungating tumor (Borrmann type 1) at the cardia (Figure 2A). The tumor measured 7 cm in the greatest diameter. The cut surface of the tumor was whitish gelatinous and the tumor involved the whole layer of the gastric wall (Figure 2B). The remaining gastric mucosa is grossly unremarkable. Microscopic feature of the resected specimen was very similar to that of the biopsy specimen except an evidence of deep invasion. Microscopically the tumor was sharply demarcated from surrounding mucosa and composed of proliferations of deceptively bland glands lined by mucin-rich columnar cells with small basal nuclei (Figure 3A). Many glands are cystically dilated especially in deep portion and their glandular lumina were filled with abundant mucin (Figure 3B). Most of glands were too bland to discriminate from benign foveolar epithelial hyperplasia (Figure 4A and 4C), but some glands were more complicated or branched with mild to moderate cellular atypia revealing increased nuclei with loss of polarity and prominent nucleoli (Figure 4B). There was no evidence of individual cell invasion into lamina propria or solid growth of tumor cells. Chronic and acute inflammatory infiltrate was heavily associated within tumor. Well formed bland glands invaded to the serosa with focal desmoplastic reaction in adjacent stroma. Vascular and perineural involvements were associated. Tumor cells metastasized to 6 out of 76 regional lymph nodes. Metastatic tumor cells within regional lymph nodes were also very bland (Figure 4D). The pathological tumor stage corresponded to stage IIIA (T3N1M0). Immunohistochemically the tumor cells revealed no overexpression of p53 protein but high Ki-67 labelling index suggesting high proliferative activity (Figure 5D). The tumor cells and intraluminal mucin were diffusely expressed MUC1 (Figure 5A) and MUC5AC (Figure 5B) suggesting gastric foveolar phenotype. MUC2 expression was only focally detected (Figure 5C). The patient has been underwent adjuvant chemotherapy. Abdominal CT taken after 12 months suggested peritoneal carcinomatosis, multiple metastatic foci in the lung, and multiple retroperitoneal lymph node enlargements. The patient has survived with an evidence of multiple distant metastases for 18 months after operation.

## Discussion

Traditionally, gastric carcinomas have been classified into two main types, the so-called intestinal and diffuse, based on the tendency toward gland-formation [1]. Recently using immunohistochemical staining techniques specific for gastric- and intestinal type mucins, the phenotype of each tumor was histologically reclassified [5,6]. Of these, MUC1 and MUC5AC are expressed in the superficial foveolar epithelium of gastric mucosa, and MUC2 is expressed in goblet cells of intestinal mucosa or intestinal metaplastic cells of stomach. Up to date several cases of EWDA of the stomach have been reported in literatures [1-3]. The reports described histologically and phenotypically heterogeneous groups of EWDA and their results were not inconsistent with the present case. Niimi *et al*, described the usefulness of p53 and Ki-67 immunohistochemical analysis for preoperative diagnosis of EWDA of the stomach. However, in the present case as well as Nokubi *et al *[3]'s case which was very similar to the present case in all respects, p53 overexpression was not observed in the EWDA. These two cases were equally associated with predominant gastric phenotype, which reflect the fact that gastric type adenocarcinoma of the stomach is less likely associated with p53 mutation pathway [6]. The immunostaining for high KI-67 was helpful to distinguish EWDA from benign foveolar epithelia in the present case.

EWDA of the stomach is a rare highly differentiated adenocarcinoma in which most of the glands are impossible to distinguish from benign foveolar glands, particularly in biopsy specimen [3]. In the present case the resected specimen EWDA showed variable histologic features area by area. Although most of tumor glands were lined by deceptively bland, mucin-rich columnar cells with basal nuclei, more atypical areas were also detected at least focally. The more atypical areas were composed of complex or haphazard arrangement of the glands as well as increased nuclei with loss of polarity and prominent nucleoli. Because the most reliable diagnostic criteria of EWDA of the stomach like adenoma malignum of uterine cervix are deeper invasion and/or metastasis, it is difficult to make a correct diagnosis in biopsy specimen. However, multifocal and repeat biopsies and careful microscopic examination can elicit the recognition of more atypical areas suggesting malignancy. The present case was misinterpreted as benign foveolar epithelial hyperplasia for the biopsy specimen, but the radiological and endoscopic findings suggested malignancy strongly. The clinicopathologic correlation is also mandatory in cases of EWDA of the stomach. First of all, to keep in mind of the entity of EWDA is essential to reach to a correct diagnosis.

## Conclusion

The clinicopathologic profiles of gastric extremely well differentiated adenocarcinoma of gastric phenotype include cardiac location, fungating gross type, very similar histology to foveolar epithelial hyperplasia, foveolar mucin phenotype, lack of p53 over expressoin and high proliferative index. In gastric EWDA of gastric phenotype, the unique criterion of malignancy is an evidence of deeper invasion. It is good reason for considering this tumor as gastric counterpart of adenoma malignum of the uterine cervix.

## Competing interests

The author(s) declare that they have no competing interests.

## Authors' contributions

WAL performed pathologic examination literature search and preparation of manuscript.
